# Heterogeneity of Functional Properties of Clone 66 Murine Breast Cancer Cells Expressing Various Stem Cell Phenotypes

**DOI:** 10.1371/journal.pone.0078725

**Published:** 2013-11-12

**Authors:** Partha Mukhopadhyay, Tracy Farrell, Gayatri Sharma, Timothy R. McGuire, Barbara O’Kane, J. Graham Sharp

**Affiliations:** 1 Department of Genetics, Cell Biology and Anatomy, University of Nebraska Medical Center, Omaha, Nebraska, United States of America; 2 Pharmacy Practice, University of Nebraska Medical Center, Omaha, Nebraska, United States of America; 3 Department of Oral Biology, Creighton University School of Dentistry, Omaha, Nebraska, United States of America; University of Wisconsin - Madison, United States of America

## Abstract

**Introduction:**

Breast cancer grows, metastasizes and relapses from rare, therapy resistant cells with a stem cell phenotype (cancer stem cells/CSCs). However, there is a lack of studies comparing the functions of CSCs isolated using different phenotypes in order to determine if CSCs are homogeneous or heterogeneous.

**Methods:**

Cells with various stem cell phenotypes were isolated by sorting from Clone 66 murine breast cancer cells that grow orthotopically in immune intact syngeneic mice. These populations were compared by *in vitro* functional assays for proliferation, growth, sphere and colony formation; and *in vivo* limiting dilution analysis of tumorigenesis.

**Results:**

The proportion of cells expressing CD44^high^CD24^low/neg^, side population (SP) cells, ALDH1^+^, CD49f^high^, CD133^high^, and CD34^high^ differed, suggesting heterogeneity. Differences in frequency and size of tumor spheres from these populations were observed. Higher rates of proliferation of non-SP, ALDH1^+^, CD34^low^, and CD49f^high^ suggested properties of transit amplifying cells. Colony formation was higher from ALDH1^−^ and non-SP cells than ALDH1^+^ and SP cells suggesting a progenitor phenotype. The frequency of clonal colonies that grew in agar varied and was differentially altered by the presence of Matrigel™. *In vivo,* fewer cells with a stem cell phenotype were needed for tumor formation than “non-stem” cells. Fewer SP cells were needed to form tumors than ALDH1^+^ cells suggesting further heterogeneities of cells with stem phenotypes. Different levels of cytokines/chemokines were produced by Clone 66 with RANTES being the highest. Whether the heterogeneity reflects soluble factor production remains to be determined.

**Conclusions:**

These data demonstrate that Clone 66 murine breast cancer cells that express stem cell phenotypes are heterogeneous and exhibit different functional properties, and this may also be the case for human breast cancer stem cells.

## Introduction

An efficient mechanism of tissue maintenance, employed by multiple normal tissues, is that of stem/progenitor cell self-renewal with regulated production of differentiated functional progeny (http://stemcells.nih.gov/info/). These processes occur in specialized microenvironments (stem cell niches), presumably to minimize the production of potentially highly proliferative abnormal cells outside the appropriate regulated site(s). A prototypical normal tissue system is the regulated production of blood and immune cells from hematopoietic stem cells (HSC) in niches in the bone marrow [1].

The notion that tumors arise from rare cells with at least some properties of stem cells has a long history. In 1974, Pierce proposed that neoplasms might be derived from stem cells whose proliferation or differentiation is dysregulated, or tumors might develop from stem/progenitor cells that are displaced and/or misregulated during development and later reactivated to form tumors [2]. For example, failure of a testis to descend increases the likelihood of its malignant transformation [3]. Dick’s laboratory [4] applied techniques for isolating and assessing functions of normal human hematopoietic stem cells to acute myelogenous leukemia (AML) cells. They showed that only a small proportion of the AML cells, not the majority population, were responsible for maintenance of the tumor. These cells were termed AML stem cells, or more generally, cancer stem cells (CSCs).

This concept has been extended to multiple other tumor types, including solid tumors such as breast cancer [5]–[9] and hepatocellular carcinoma [10]. However, this generalization is not without controversy [11]–[14]. One definition offered for human CSC is that they have the ability to form tumors in immunodeficient mice [15]. However, Kelly and colleagues [16] noted that only rare human lymphoma cells meet this criterion. In contrast, in an Eµ myc mouse syngeneic system, 10% or more of lymphoma cells were tumorigenic. They suggested the apparent rarity of stem cell-like cells of human lymphomas that form tumors in immunodeficient mice might simply be consequence of mismatch(es) between the human cells and the mouse microenvironment. It has also been suggested that this limitation might be partly responsible for the high Phase II attrition rate seen in oncology trials [17], [18].

An inherent assumption in many studies of both normal stem cells and CSC is that a homogeneous population is being analyzed, and this is depicted on lineage diagram by a single cell. However, in the case of leukemia stem cells, the reality has become more complex with greater heterogeneity and dependence on microenvironment than previously thought [19]. In reality, if normal human hematopoietic stem cells (HSCs) from the same tissue sample, but with different phenotypes *e.g.,* side population (SP) versus CD34^high^ cells, are analyzed for production of human cells in immunodeficient mice, differences are observed [1]. This suggests that there is a hierarchy of functional properties expressed by normal stem/progenitor cells. Even using different expression levels of a specific stem cell marker to isolate normal HSC, *e.g.*, SP, produces cells with functional heterogeneities [20]. Whether this is also the case for CSC has been addressed [7], [8], [13], [21] but not fully evaluated, although it is important because it could explain differences in CSC properties that have been reported by different groups who generally only employ a single isolation technique that often differs between groups *e.g*., Aldefluor vs. SP cells [22], [23].

Consequently, the present study was devised to determine if breast cancer cells that met the definition of CSC, based on expression of different specific stem cell markers, were functionally homogeneous or heterogeneous, based not only on phenotype but also on *in vitro* assays of stem cells (mammosphere formation) or limiting cell dilution analysis of *in vivo* tumorigenesis. Additional characteristics including cell growth, colony formation, expression profiling, response to growth factor rich matrix (Matrigel™), and cytokine production were evaluated. To avoid the criticisms applied to prior studies *e.g.*, [16]; an orthotopic syngeneic murine model that mimics the growth of human breast cancer cells in immune intact individual, the Clone 66 model [24], [25] was employed. This model also has the advantage that it contains cells that express various putative CSC markers (SP, CD44^high^CD24^low/neg^, ALDH1^+^, CD34^high^, CD133^high^, CD49f^high^ and others); so that all these cell types can be legitimately compared for functional properties.

## Materials and Methods

### Cell Line

Clone 66 (Cl66), a murine adenocarcinoma cell line, derived from a spontaneously arising mammary tumor in a Balb/cfC3H female mouse originally described by Dexter and colleagues [24], was generously made available by Dr. James Talmadge of the University of Nebraska Medical Center, Omaha, Nebraska. This cell line has been described in a previous publication [25] and grows and metastasizes in an orthotopic immune intact syngeneic model in a manner similar to triple negative human breast cancer.

### Cell Culture

A limited number of passages of parental Cl66 were maintained, tested to be free of mycoplasma contamination, and cryopreserved. All experiments were performed with cells at less than 20 passages after receipt. The DMEM (Dulbecco’s Modified Eagles Medium) media (Invitrogen, Carlsbad, CA, USA) supplemented with 10% fetal bovine serum (FBS), 100 U/ml penicillin, 100 µg/ml streptomycin, 2 mM L-glutamine, 10 mM HEPES (pH 7.4), 1× MEM vitamin solution, 1× Basal Medium Eagle amino acid solution, and 1× Modified Eagles Medium (MEM) non-essential amino acids were used for maintaining Cl66 cells. All materials were purchased from Gibco-BRL (Gibco-BRL, Grand Island, New York, USA).

### Animals

Female Balb/c mice, 6–8 weeks of age, were purchased from Jackson Laboratory (Jackson Laboratory, Bar Harbor, ME, USA). Mice were maintained in micro-isolators in the USDA (U.S. Department of Agriculture)/AALAC (American Association of Laboratory Animal Science) accredited facility at the University of Nebraska Medical Center on a 12 hours light, 12 hours dark cycle. The mice were allowed food and acidified water ad libitum.

### Fluorescence Activated Cell Sorting (FACS) Analysis

#### Side population analysis

Side population analyses were performed following the method described previously [23]. Briefly, 1×10^6^ cells were incubated overnight (or at least for 2 hours) at 4°C in a small volume (1.0 ml) of pre-warmed Hoechst IMDM (Iscove’s Modified Dulbecco’s Medium). In staining media the concentration was (1µg/ml). Adding an appropriate amount of Hoechst 33342 dye (Sigma-Aldrich, St. Louis, MO, USA), the specimen being stained was incubated at 37°C for 30 minutes. After incubation, cells were kept on ice followed by flow analysis. **Antibody staining:** Following enzymatic/mechanical disaggregation Cl66 cell populations were filtered through a 40 µm nylon mesh (BD Biosciences, CA, USA) to ensure single cellularity and re-suspended in ice-cold PBS (containing 1% BSA) to a density of 10^6^ cells/200 µL and incubated with the following antibodies: PE- conjugated anti -CD34 (12-0341-81, 1∶100), AF488- conjugated anti -CD133 (53-1331-80, 1∶100), PE- conjugated anti -CD49f (12-0485-82, 1∶100) were purchased from eBioscience (eBioscience, San Diego, CA, USA). FITC- conjugated anti -CD44 (553133, 1∶100), and PE- conjugated anti -CD24 (553262, 1∶100) were purchased from BD Biosciences (BD Biosciences, CA, USA). Cells were analyzed using a BD FACSAria. Background signals due to autofluorescence were eliminated using normal tissues/cells. The remaining positive events (background) were subtracted from the experimental samples. **ALDH1 activity assay:** ALDH1 activity in cells was detected with an Aldefluor Kit, according to manufacturer’s instructions (Stem Cell Technologies, Vancouver, BC, Canada) followed by FACS analysis. DEAB (ALDH1 inhibitor) treated cells were used to set background and also to correctly identify ALDH1 fluorescence. **Tissue preparation:** at necropsy, tumors to be analyzed were removed, placed in sterile medium and minced with scissors. The tissue was then transferred to a 15 ml conical tube and repeatedly aspirated through syringes with successively smaller sized needles until a single cell suspension had been achieved. The cells were washed and analyzed in HPBS (1% FBS) and stained as above. The number of positive cells was calculated based on the assessment of fluorescence intensity greater than that of background with tissue from normal control animals.

### Sphere Formation Assay

Sphere formation assays were performed as described previously with modifications [26]. Briefly, Single-cell suspensions were re-suspended at a density of 1000 cells/ml in ultralow attachment 6-well or 12-well dishes (Corning Incorporated, NY, USA) in DMEM/F-12 (Invitrogen, CA, USA) containing 5 µg/mL insulin, 0.5 µg/mL hydrocortisone, 25 ng/mL EGF, 25 ng/mL bFGF, Heparin 4 µg/mL (0.5 U/mL), 1% BSA and Gentamycin sulfate (Gibco-BRL, NY, USA). Cultures were fed weekly and tumor sphere formation was monitored each week. Tumor spheres were disaggregated periodically by 5 min Accutase treatment (Stem Cell Technologies, Canada) followed by mechanical disaggregation with a sterile Pasteur pipette. Phase-contrast images were obtained under 40× magnification to visualize the morphology of sphere, and all spheres were counted, plotted and presented as histogram. Each experiment was performed in triplicate. More than two independent experiments were performed.

### Assay for Anchorage Independent Growth in Soft Agar

Anchorage-independent growth assays were performed as described previously with reduced cell numbers [27]. Briefly, 5×10^2^ cells of various populations without or with stem phenotypes were plated in 6-well plates in 1.5 mL of 0.35% agarose (Sigma-Aldrich, MO, USA) in DMEM media on top of a bottom layer of 0.5% agarose in DMEM media. Plates were incubated for 2 weeks. Phase-contrast images were obtained under 100× magnification to visualize the morphology of agar colonies, and colonies were counted, plotted and presented as histogram. Each experiment was performed in triplicate. At least two independent experiments were performed.

### Growth Kinetic Studies

Various sorted cells were cultured overnight to allow cells to recover from the stress of sorting. Growth kinetics and population doubling time of various cell populations with or without stem phenotype were determined as described previously [28]. Briefly, for growth curves, cells were seeded at 1×10^4^ cells/well in 6-well-plate in triplicate. Viable cells of different populations in each well of the 6-well plates were counted for 7 days by a viable cell counter (ViCell Coulter counter, Beckman Coulter, Inc., CA, USA). Population doubling times of various cell populations with or without stem phenotype were calculated from the number of cells growing in the log phase (96–144 h) and using the formula: T_d_ = 0.693 t/ln (N_t_/N_0_), where t was time (in hour), N_t_ was the cell number at time t, and N_0_ was the cell number at the initial time.

### Colony Forming Assays

Colony forming assays were performed as described earlier [29]. Briefly, the colony-forming efficiency of various cell populations with or without stem phenotypes was examined 14 days after plating 250 cells plated in quadruplicate, by staining with crystal violet (Sigma-Aldrich, MO, USA). Colonies of >50 µm in size were counted using quantity One software (Bio-Rad, Richmond, CA, USA). Results are presented as an average of 3 independent experiments.

### *In vivo* Tumorigenesis in Syngeneic Balb/c Mice

Cell populations without or with stem cell phenotypes were harvested using 0.05% trypsin-EDTA (Gibco BRL, NY, USA) washed twice and suspended in HBSS (Gibco BRL, NY, USA), without serum, immediately prior to mixing with Matrigel™ (BD Biosciences, San Jose, CA, USA). The cells were adjusted to deliver the desired number of cells in a total volume of 0.05 ml with 1∶1 dilution of Matrigel™. The cell suspensions were injected into an intact inguinal mammary fat pad. In first set of experiments, 6000, 3000, 1000 cells were transplanted in triplicate into mammary fat pads. Tumors generated from each group (of 6000, 3000, and 1000 cells) were dissociated into single cell suspensions, analyzed for stem cell phenotypes, cultured for two weeks and re-transplanted using 1000 cells of the pooled population of each group. In second set of experiments, 1000, 500, 200 cells were transplanted into mammary fat pads. Again, tumors generated from each group of 1000, 500, and 200 cells were dissociated into single cell suspensions, cultured for two weeks and re-transplanted using 500 cells of the pooled population of each group. In a third set of experiments, 50, and 25 SP cells were transplanted into mouse mammary fat pads. Initially, low numbers of tumors without major differences in tumorigenic potential of ALDH1^+^ and ALDH1^−^ cells were observed. Consequently, 24000, 10000, and 6000, cells of ALDH1^+^ and ALDH1^−^ cells were transplanted, in triplicate, into mammary fat pads of mice. Mice were monitored on alternate days for the development of tumors at the injection site and the frequencies of tumor noted. The tumor size was measured (W×H×L) and the volumes were calculated.

### Ethics Statement

All experiments were carried out in accordance with the recommendations in the Guide for the Care and Use of Laboratory Animals of the National Institutes of Health. The protocol was approved by the Committee on the Ethics of Animal Experiments of the University of Nebraska Medical Center, Omaha, Nebraska (Permit Number: 11-004-07-FC). All transplantations were performed under isoflurane anesthesia. Finally, mice were euthanized according to IACUC (Institutional Animal Care and Use Committee) guidelines and excised tumors were subjected to histological analyses.

### Histology

Tumors recovered, at necropsy, were fixed in 10% buffered formalin and processed to prepare routine procedures for paraffin sections. Briefly, following fixation, the tissue was dehydrated using a graded series of alcohol baths beginning with 50% and progressing to100%. The tissue was then cleared with xylene, infiltrated and embedded in paraffin. Five micron tissue sections were cut using a Leica rotary microtome and stained with hematoxylin and eosin for morphological analysis. **Hematoxylin and eosin (H&E) staining:** H&E staining was performed following the methods described previously [30]. Slides were deparaffinized, rehydrated and stained with Harris hematoxylin and eosinY solutions (Surgipath, Richmond, IL, USA). After staining, the sections were dehydrated and coverslipped using Limonene Mount (Sigma-Aldrich, MO, USA). All histological material was examined and photographed using a Neurolucida virtual microscopy scanning system (MBF Bioscience, VT, USA) attached to Zeiss Axioskop2. The tumor types were characterized and evaluated for hypoxic areas, blood vessel densities (BVD), and evidence of secretory product.

### Cytokine Assays

Unsorted Cl66 cells at a density of 10^5^ per 6 cm dish were grown in culture with or without MS-5 stromal cells and supernatants were harvested on day 3. Supernatants were centrifuged at 14,000×g for 5 minutes, and transferred into a clean test tube. Protein concentrations were determined using a BIO-RADD/C (Bio-Rad, Richmond, CA, USA) protein estimation kit. Levels of a wide range of inflammatory/angiogenic cytokines and chemokines were measured using a mouse Proteome Profiler Array (R&D Systems, MN, USA) following the manufacturer’s procedure. Briefly, 50 µL of cell culture supernatants were prepared for addition to antibody pre-spotted nitrocellulose strips. Antibodies were spotted in duplicate on each nitrocellulose strip. After binding the complementary cytokine/chemokine during an overnight incubation, strips were treated with streptavidin-HRP containing buffer, followed by treatment with chemiluminescent reagent. The enhanced chemiluminescence signals were recorded using a light-sensitive film (GeneMate Blue Lite Autorad Film, BioExpress, Kaysville, UT, USA), which was scanned to determine the relative intensity matrix. Pixel density associated with the chemiluminescent signal measured was converted to intensity 0 to +4. The average value for each cytokine, after subtracting background values, was graphed as histograms that allowed comparison of cytokine production under various experimental conditions.

### Statistical Analyses

Data were analyzed using two-tailed Student’s *t*-tests in Excel and two-tailed Fisher’s exact tests where appropriate, using Microsoft Excel 2010. P<0.05 was considered statistically significant. Flowjo V4 (Tree Star, Inc., Ashland, OR, USA) was used for FACS data analysis.

## Results

### Cl66 Murine Breast Cancer Cells Comprised Distinct Cell Subpopulations with or without Stem Cell Phenotypes

**Figure 1:**
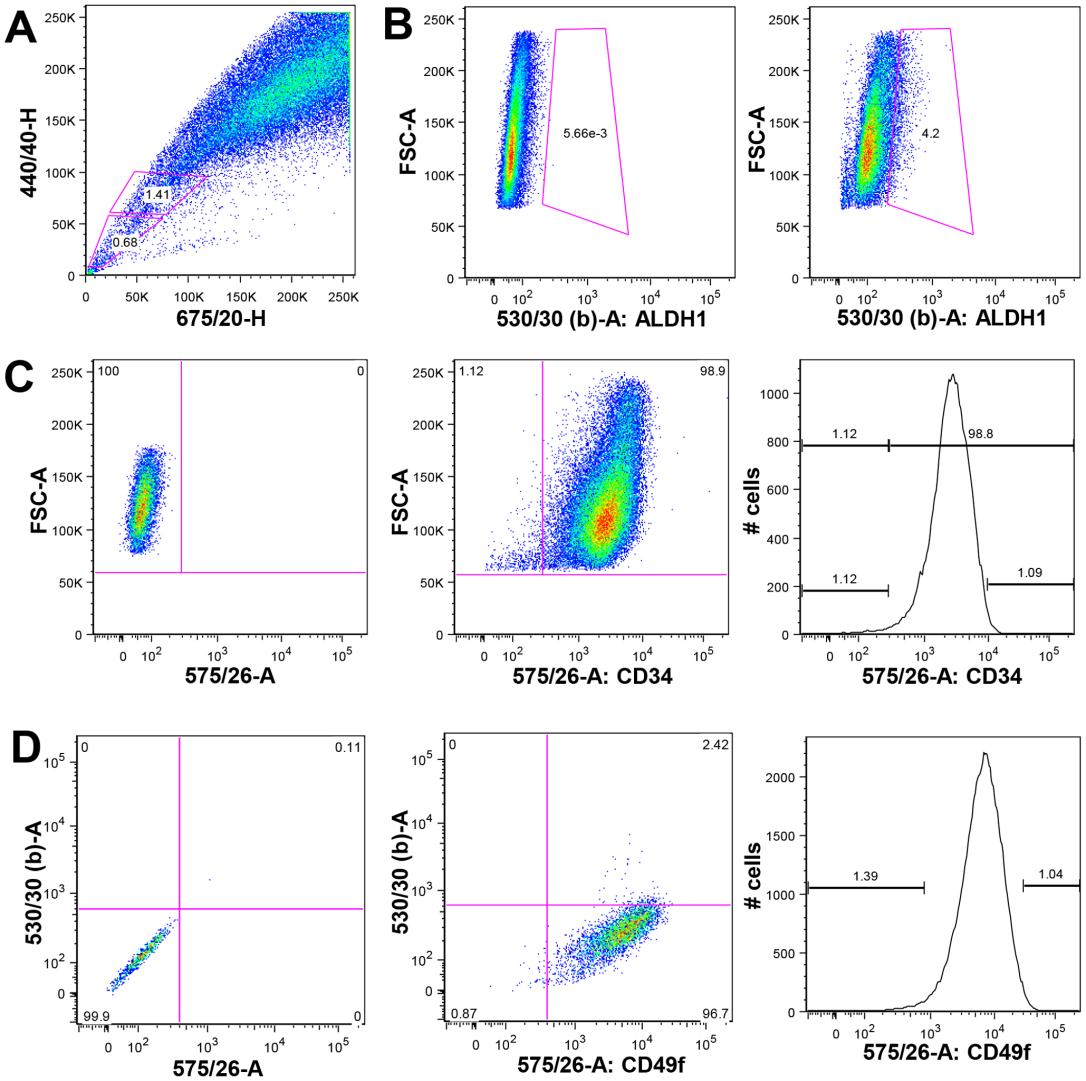
Expression of putative stem cell markers in Clone 66 murine breast cancer cells. (**A**) After staining Cl66 cells with Hoechst 33342 dye followed by FACS analysis, we detected 1.23% ±0.95% cells were SP cells (n = 3). (**B**) When Cl66 cells were FACS analyzed after Aldefluor treatment with or without DEAB (ALDH1 inhibitor), we found approximately 4.16% ±3.26% (n = 3) cells were ALDH1^+^. (**C**) After staining with anti-CD34 antibody followed by FACS analysis, we identified 90% ±13%, n = 4 cells were positive for CD34. Cells expressed highest levels of CD34 (CD34^high^) and lowest levels of CD34 (CD34^low^) were selected and sorted for this study. (**D**) After staining with anti-CD49f antibody followed by FACS analysis, we identified 99.47% ±0.21% (n = 3) cells were positive for CD49f. Cells with the highest levels of CD34 expression (CD34^high^) and lowest levels of CD34 expression (CD34^low^) were selected and sorted for this study. For the SP analysis, an X-cite LightWave air-cooled 20 mW UV laser at 354 nm (made by JDS Uniphase) was used. For the ALDH1, PE, FITC and Alexa488 analysis, a Saphire air-cooled 100 mW blue laser at 488 nm (made by Coherent) was used. Average data from more than two independent assays are shown. n = number(s) of assay(s) performed.

We employed Cl66 cells as this is an orthotopic model that grows in mammary fat pad and metastasizes in immune intact mice [25]. This model replicates the growth and metastasis of human breast cancer. In a preliminary screening of breast cancer cell populations, isolated using FACS sorting from Cl66, we observed the presence of varying proportions of SP, CD44^high^CD24^low/neg^, ALDH1^+^, CD34^high^, CD133^high^, and CD49f^ high^ cells (Table 1, Figure 1). There was about a 3000 fold variation in the proportion of cell populations with “stem cell” phenotypes (Table 1). A cell population with CD44^high^CD24^low/neg^ phenotype was present at 0.03% ±0.01% in Cl66 and it was challenging to isolate sufficient cells for functional studies. CD133^high^ cells were also present in Cl66 cells but the flow profile of these cells indicated that adequate separation by sorting would be challenging. Consequently, cells with this phenotype were excluded from this study. From the results presented in Table 1, we concluded that Cl66 murine breast cancer cells expressing various stem phenotypes were likely heterogeneous based on phenotypes. This prompted questions as to potential heterogeneities of their functional properties. We performed additional experiments in order to test this hypothesis. In the interest of brevity only the most informative data are presented.

**Table 1:** Various cell populations (%) with stem cell phenotypes were evaluated in Clone 66.

References	Tumor type	Putative stem cell markers usedfor sorting	Average cell populations (%) present in Clone 66 in this study
[54]	Acute myeloid leukemia	CD34^high^	90%±13%, n = 4
[57]	Brain cancer	CD133^high^	0.32±0.28%, n = 2
[5]	Breast cancer	CD44^high^CD24^low/neg^	0.03%±0.01%, n = 2
[23]	Breast cancer	SP	1.23%±0.95%, n = 3
[22]	Breast cancer	ALDH1^+^	4.16%±3.26%, n = 3
[58]	Breast cancer	CD49f^high^	99.47%±0.21%, n = 3

### Various Cell Populations with Stem Phenotypes Generated Tumor Spheres at High Efficiency

**Figure 2:**
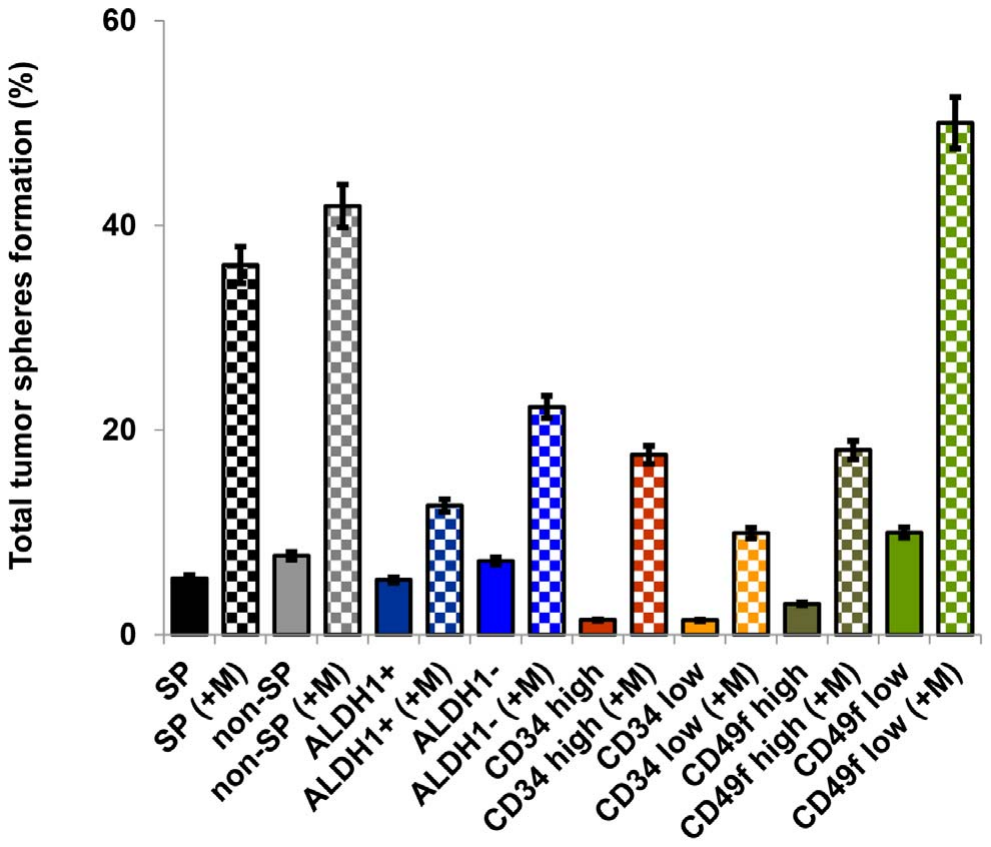
Evaluation of self-renewing cells in sorted cell populations of Cl66 murine breast cancer cells. Tumor spheres were obtained by culturing sorted cells from Cl66 murine breast cancer cells in tumor sphere medium containing EGF and bFGF. Cells were plated at a density of 1000 cells/ml. Percent sphere formation was calculated and plotted against stem or non-stem phenotype with (+M) or without Matrigel™. Cell population with the SP phenotype showed formation of larger spheres than cells with other phenotypes. Sphere forming efficiency of SP and ALDH1^+^ phenotype was higher (4–5%) than CD34^high^ and CD49f^high^, which were 2–4%. Formation of spheres was observed from cell populations with stem and non-stem cell phenotypes. The only exception was cell population with CD49f^low/neg^ phenotype (a purported non-stem phenotype), which formed more spheres than the CD49f^high^ cell population. Addition of Matrigel™ increased the numbers of spheres of cell populations with stem and non-stem cell phenotypes. Data are the average of two independent experiments.

The use of the mammosphere assay to evaluate the presence of stem cells in a population of mammary epithelial cells (MECs) was previously validated by the capacity of a single murine mammosphere to regenerate an entire mammary ductal tree when transplanted into a cleared mouse mammary stromal fat pad [31]. Here, we sought to detect the frequency of sphere formation among various cell populations with or without stem phenotypes such as, SP or non-SP, ALDH1^+^ or ALDH1^−^, CD34^high^ or CD34^low^, and CD49f^high^ or CD49f^low/neg^ cells. These various cell populations with or without stem phenotypes were analyzed for tumor sphere formation [26], [32]–[37] in serum free medium supplemented with bFGF and EGF. Cells with stem phenotypes quickly developed tumor spheres, while we observed low sphere formation when the cell populations with non-stem phenotype were grown under the same culture conditions (Figure S1). Cells with the SP phenotype showed formation of larger spheres than cells with other phenotypes. Sphere forming efficiency of SP and ALDH1^+^ phenotype was 4–5% whereas sphere forming efficiency of CD34^high^ and CD49f^high^ was 2–4% (Figure 2). Addition of Matrigel™ (a source of growth factors?) increased the size of spheres with SP, ALDH1^+^, CD34^high^, and CD49f^high^ when compared with their respective non-stem cell populations. The only exception was CD49f^low/neg^ cell population (a purported non-stem phenotype), which formed more spheres than the CD49f^high^ cell population, suggesting that this phenotype does not select for sphere forming cells. Secondary sphere formation was observed only with SP, CD34^high^, CD49f^high^ and CD49f^low^ cells (data not shown). Very limited or no secondary sphere formation was observed with ALDH1^+^, ALDH1^−^ and non-SP cells. Secondary sphere formation was much higher with SP than CD34^high^ cells. This indicated differential self-renewal of sphere forming cells with a SP phenotype under *in vitro* conditions. Consistent with the primary sphere formation, CD49f^high^ and CD49f^low^ cells formed secondary spheres in significant numbers. Overall, these data indicate that sphere formation by ALDH1^+^, SP, CD34^high^, and CD49f^high^ cell populations was heterogeneous, suggesting differences in self-renewal.

### Cell Populations with Stem Phenotypes Exhibited Reduced Proliferation, Lower Colony Formation and Enhanced Agar Colony Formation

**Figure 3:**
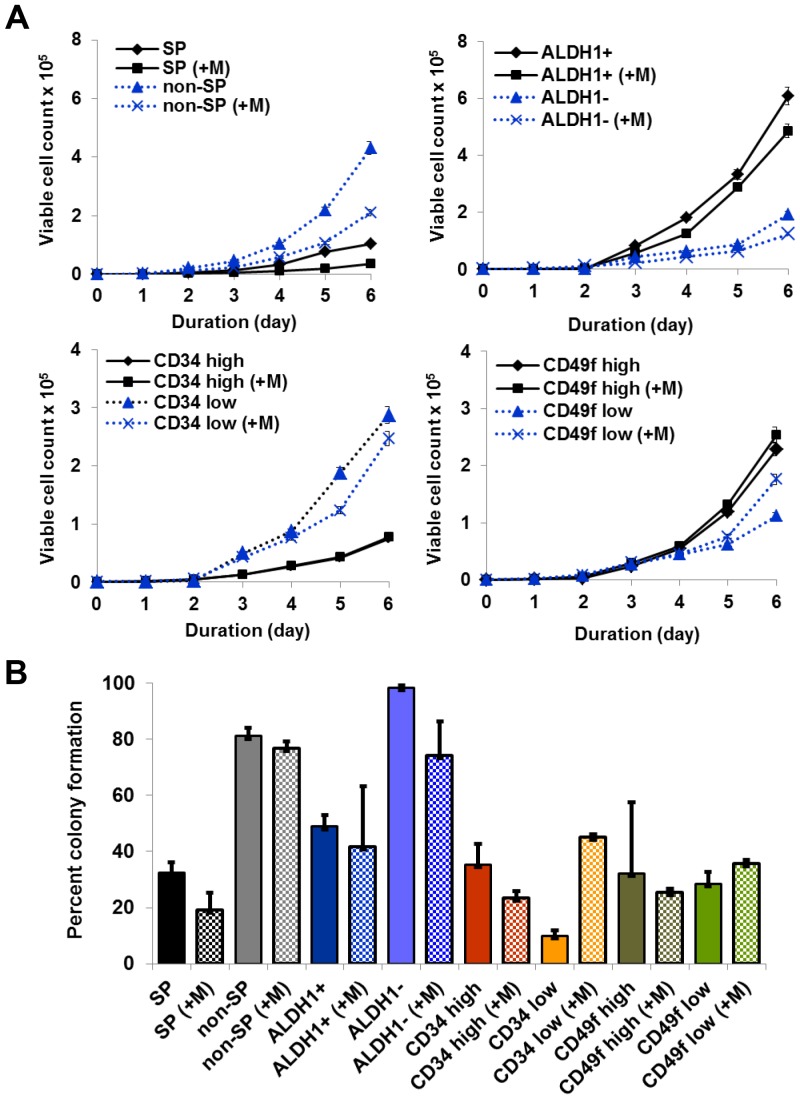
Cell populations with stem phenotypes exhibited reduced proliferation and lower colony formation. (**A**) In proliferation analyses, when the number of cells was plotted against the incubation period (days), the proliferation rate was much higher in ALDH1^+^, non-SP (non-side population), CD34^low^, and CD49f^high^ than their respective counterparts. Matrigel™ only favored the proliferation of the CD49f^low/neg^ population. Population doubling times of ALDH1^+^and CD49f^high^ cells (17±0.2, 20±0.4 hours) was less than ALDH1^−^ and CD49f^low^ cells (21±0.26, 24±0.41 hours) when calculated from the number of cells growing in log phase (day 2 to 6) using the formula, T_d_^ = ^0.693 t/ln (N_t_/N_0_). CD34^high^ showed higher (25±0.43 hours) population doubling time than CD34^low^ (20±0.33 hours). With Matrigel™ indicated by +M. (**B**) When analyzed for colony formation efficiencies, cell populations with ALDH1^−^, non-SP, CD34^high^, CD49f^high^ phenotypes showed higher colony forming efficiency than their respective counterparts. The CD49f^high^ and CD49f^low/neg^ cells did not show major differences in colony forming efficiencies, suggesting that this phenotype might not be a suitable phenotype to differentiate stem and non-stem phenotype, at least under *in vitro* culture conditions. Matrigel™ increased colony formation only for the CD34^low^ population. With Matrigel™ indicated by +M.

**Figure 4:**
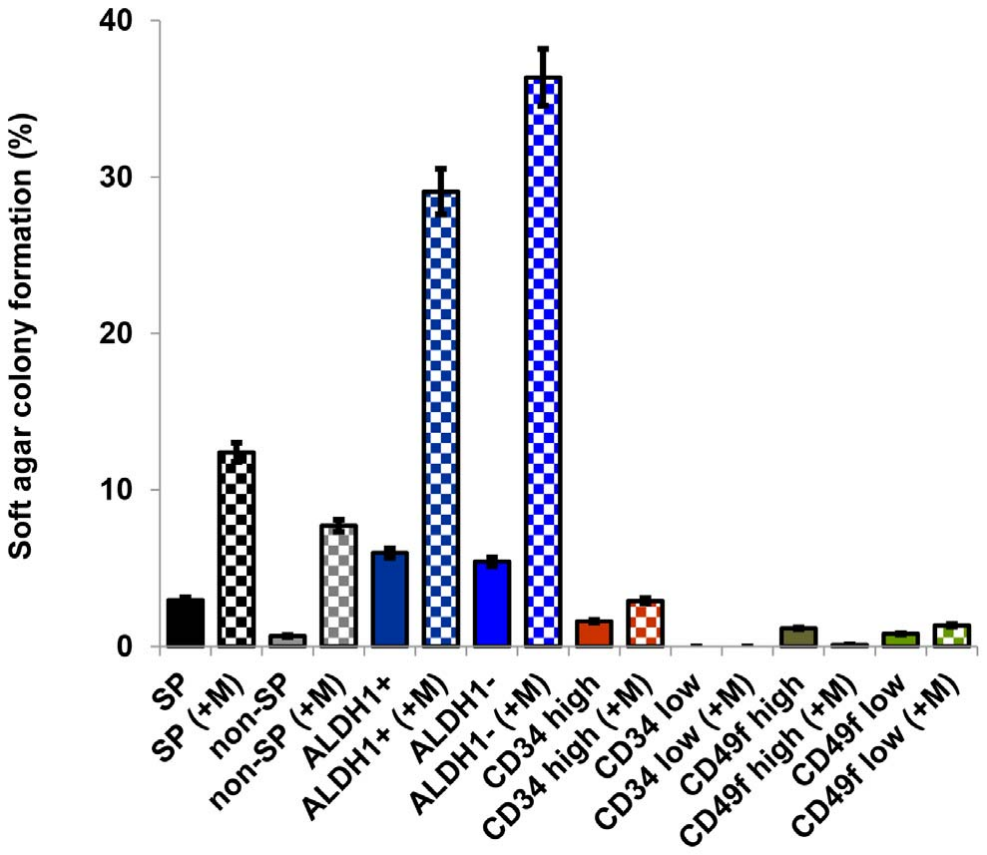
Cell populations with stem phenotypes exhibited enhanced agar-colony formation. Agar colonies were obtained by culture of sorted cells from mouse mammary carcinoma Cl66 cells cultured in soft-agar containing complete DMEM media. Stem-type cells formed higher numbers of agar colonies than non-stem type cells. ALDH1^+^ and ALDH1^−^ formed more agar colonies than SP cells and SP cells formed more agar colonies than non-SP cells. Soft agar colony formation was much lower in CD34^high^ and CD49f^high^ cell populations. Matrigel™ increased the size of agar colonies of all cell populations, indicating that all cell populations responded to the growth factors present in Matrigel™. With Matrigel™ indicated by +M. All data presented are the average of 2 independent experiments.

Proliferation, a major property of cancer cell populations, is mostly exhibited by progenitor and transit amplifying cells, whereas stem cell populations are postulated to exhibit quiescence; so we sought to investigate the proliferation of various cell populations with or without stem cell phenotypes. Proliferation analysis indicated that the proliferation rate was much higher in ALDH1^+^, non-SP, CD34^low^, and CD49f^high^ than their respective counterparts (Figure 3A). Population doubling times of ALDH1^+^, and CD49f^high^ (17 hours, and 20 hours) was less (p = 0.0004, p = 0.01) than ALDH1^−^, and CD49f^low^ (21 hours, and 24 hours). The population doubling time of CD34^high^ cells (25 hours) was higher (p = 0.0001) than CD34^low^ (20 hours). This data correlated well with the proliferation data. Although the growth of SP cells was much lower than that of the non-SP population, cell death associated with the non-SP population made it challenging to determine actual population doubling times. Potentially, the clastogenic effects of the Hoechst dye, which unlik**e** SP cells, was not excluded from non-SP cells, might have contributed to cell death in this population. Matrigel™ favored the proliferation of the CD49f^low/neg^ population (Figure 3A). These results suggested that SP, CD34^high^, and CD49f^low/neg^ have higher frequencies of stem cell populations. Although ALDH1^−^ cells showed less proliferation and thereby might have higher frequencies of stem cell populations, this finding is not supported by the published literature [22] and might need further investigation. When we analyzed colony formation efficiencies, cell populations with ALDH1^−^, non-SP, and CD34^high^ phenotypes showed higher (p = 0.002, 0.0006, 0.005) colony forming efficiency than their respective stem cell phenotype positive counterparts (Figure 3B). The CD49f^high^ and CD49f^low/neg^ cells did not show major differences in colony forming efficiencies (p>0.05) suggesting that this phenotype might not be a suitable phenotype to differentiate stem and non-stem cell populations under in *in vitro* culture conditions. Matrigel™ increased colony formation only for the CD34^low^ population. These results suggest that ALDH1^−^, non-SP, CD34^high^, and CD49f^high^ cells have a greater proportion of progenitor cells than their stem cell phenotype counterparts. Almost all cell populations with stem or non-stem phenotype formed agar colonies (Figure S2) without or with Matrigel™. Calculated total colonies were found to vary in numbers (Figure 4). Cell populations with stem cell phenotypes formed higher numbers of agar colonies than cell populations with non-stem phenotypes. ALDH1^+^ and ALDH1^−^ formed more agar colonies than SP cells and SP cells formed more agar colonies than non-SP cells (Figure 4). Soft agar colony formation was much lower in CD34^high^ and CD49f^high^ cell populations. Matrigel™ increased the size of agar colonies of all cell populations, indicating that all cell populations responded to the matrix or growth factors present in Matrigel™. The agar colony formation data suggested that the oncogenic phenotype is more highly represented in ALDH1^+^, SP, and CD34^high^ cells than their non-stem cell counterparts. CD49f ^low/neg^ cells showed more agar colony formation (or oncogenic potential) than CD49f^high^ suggesting that this phenotype is inadequate for distinguishing stem versus non-stem phenotype. Indeed, CD49f ^low/neg^ cells might be more oncogenic and enriched with stem cells, but this needs further confirmation. Consequently, cells with this phenotype were not evaluated further. The differences of functions of these cell populations might be an outcome of transcriptional changes or post-translational (microRNA) differences between the cell populations with a stem cell phenotype, as observed in other systems [38], [39] and might reflect soluble factor production [40]. This possibly requires further evaluation.

### Cell Populations with Stem Phenotypes have a Higher *in vivo* Tumorigenic Potential

After performing *in vitro* functional studies, without and with Matrigel™, we sought to investigate the tumorigenic potential of isolated cell populations with or without stem phenotypes using mammary fat pad transplantation that provides a suitable stromal microenvironment for tumor growth. Based on the results of the *in vitro* sphere forming assays, which indicated that the SP and ALDH1^+^ possessed stem cell activities and non-SP and ALDH1^−^ cells were less potent and given that both CD49f^high^ and CD49f^low^ cells had similar sphere forming activities; and CD34^high^ and CD34^low^ cells had less sphere and agar colony forming activities than SP and ALDH1 phenotypes, the focus of the *in vivo* tumorigenic assays emphasized a comparison of SP to non-SP cells and ALDH1^+^, and ALDH1^−^ cells.

**Figure 5:**
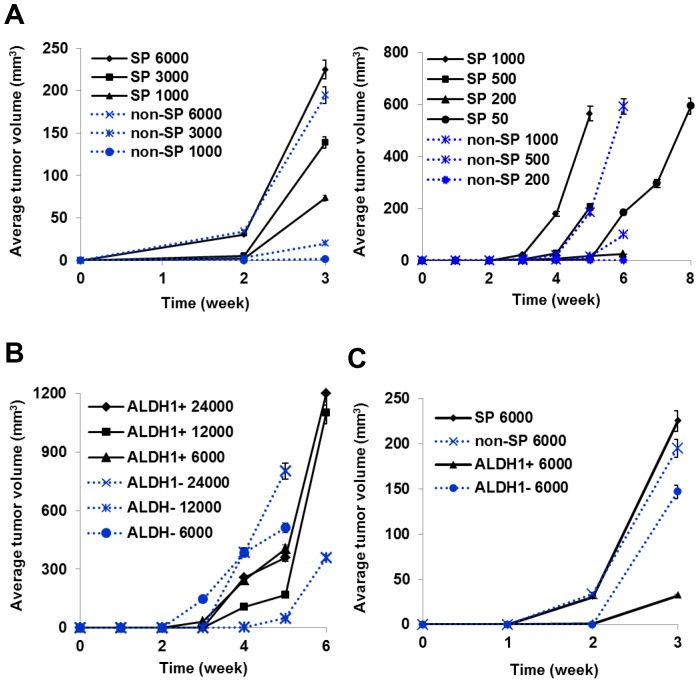
SP cell populations with stem phenotypes have higher tumorigenic potentials. Various cell populations with stem phenotypes were transplanted orthotopically at different dilutions (24000, 10000, 6000, 3000, 1000, 500, 200, 50, or 25 cells/fat pad) with 1∶1 Matrigel™ into mouse mammary fat pads. Tumor volumes were calculated every alternate day for 6 weeks. When tumor growth was below 10 mm^3^, mice were necropsied following guideline and tumors were excised and analyzed. (**A**) Results indicated that fewer SP cells were needed to form tumors than ALDH1^+^cells, suggesting heterogeneities in tumorigenicity. A characteristic that might be related to these heterogeneities was levels of cytokines/chemokines produced by the differing cell populations. (**B**) ALDH1^+^ and ALDH1^−^ did not show consistent differences in tumor formation. (**C**) Volumes of tumors formed from 6000 cells of SP, non-SP, ALDH1^+^ and ALDH1^−^ cell populations showed that SP grew faster than non-SP, ALDH1^+^ and ALDH1^−^ cells.

Various cell populations with or without stem phenotypes were transplanted at limiting cell dilutions (24000, 10000, 6000, 3000, 1000, 500, 200, 50, or 25 cells/fat pad) with 1∶1 Matrigel™ orthotopically into multiple mouse mammary fat pads of two groups of female Balb/c mice (n = 3). SP cells (1000, 3000, and 6000 cells) produced detectable tumors at week 3, while tumors resulting from non-SP cells (≤3000 cells) were detectable only after 5 weeks (Figure 5A). However, 6000 cells of SP and non-SP produced almost similar tumor formation at week 2–3 indicating quantitative rather than qualitative differences in tumorigeneicity. Results indicated that fewer SP cells (<100 cells/fat pad) were needed to form tumors than ALDH1^+^ cells (>1000 cells/fat pad) suggesting heterogeneities in tumorigenesis (Table 2). Further, ALDH1^+^ and ALDH1^−^ did not show consistent differences in tumor formation (Figure 5B; Table 2), which suggests that ALDH1^+^ and ALDH1^−^ cell populations might have lower frequencies of stem-type cells or differed in factor(s) production. Cell populations with an SP phenotype but not with non-stem phenotype (non-SP) formed tumors in all mice receiving 200 or more cells suggesting a higher frequency of stem type cells in the SP population. When compared, volumes of tumor formed from 6000 cells of SP, non-SP, ALDH1^+^ and ALDH1^−^ cells, showed SP tumors grew fastest (Figure 5C). Consequently, it might be possible that the environment of the mammary fat pad favors enhanced proliferation of SP cells when compared with non-SP, ALDH1^+^ and ALDH1^−^ cells.

**Table 2:** Formation of tumors by cell populations with stem/non-stem cell phenotypes.

Cell phenotypes	# of cells transplanted to 3or 4 separate fat pads/mouse	% mice with tumor	Cell phenotypes	# of cells transplanted to 3or 4 separate fat pads/mouse	% mice with tumor
SP	6000	100	ALDH1^+^	24000	50
SP	3000	100	ALDH1^+^	10000	50
SP	1000	100	ALDH1^+^	6000	33
SP	500	100	ALDH1^+^	3000	0
SP	200	100	ALDH1^+^	1000	0
SP	50	67			
SP	25	0			
Non-SP	6000	100	ALDH1^−^	24000	50
Non-SP	3000	100	ALDH1^−^	10000	25
Non-SP	1000	100	ALDH1^−^	6000	25
Non-SP	500	100	ALDH1^−^	3000	33
Non-SP	200	0	ALDH1^−^	1000	0

**Figure 6:**
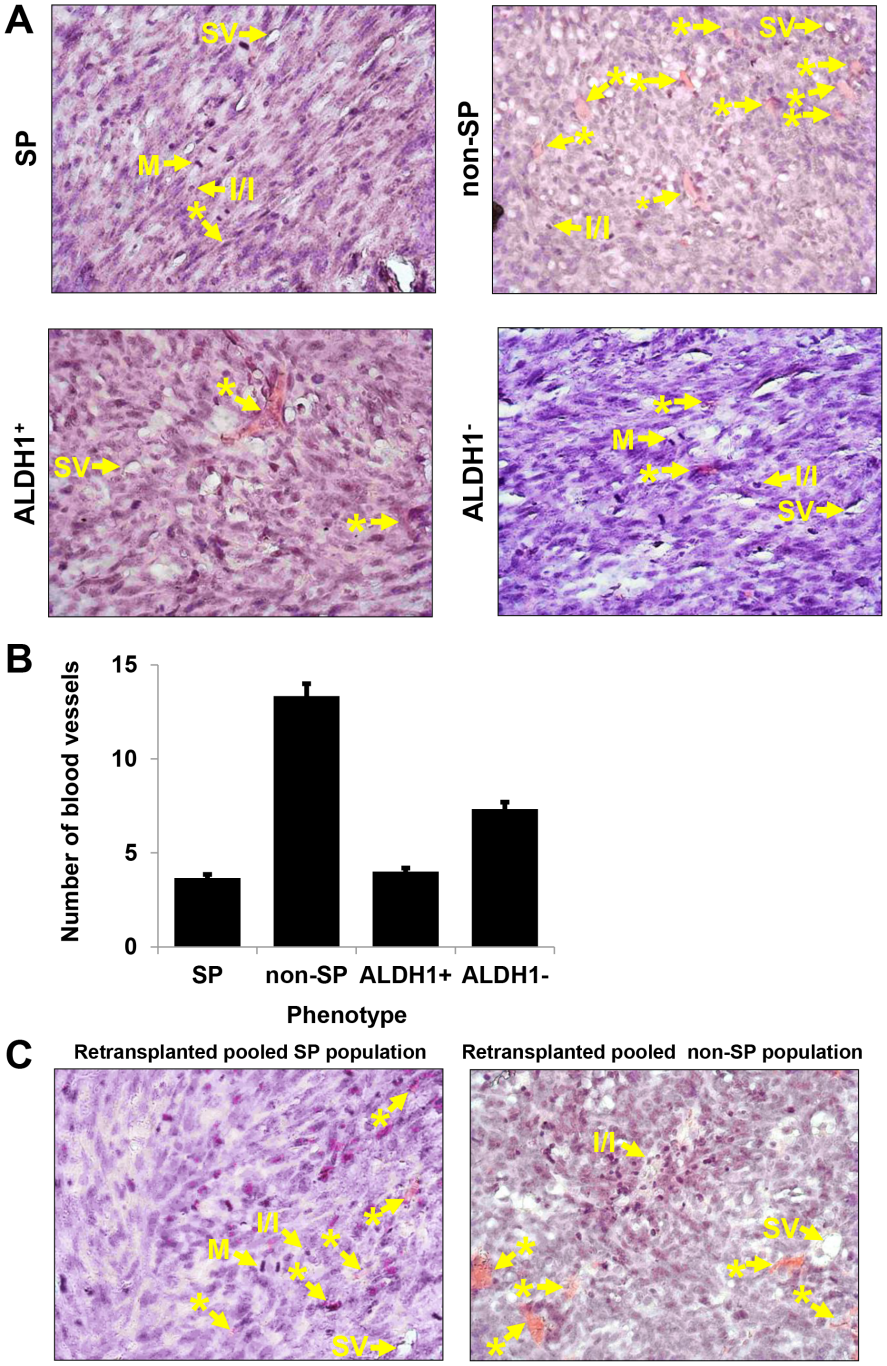
Histological analyses of tumors generated in mammary fat pads of Balb/c mice. Tumors were generated from SP, non-SP, ALDH1^+^, or ALDH1^−^ cells transplanted orthotopically in mammary fat pads of Balb/c mice. (**A top panel**) H & E stained tumor sections generated from SP and non-SP cells demonstrated the presence of blood vessels, inflammatory/immune cells with heterochromatic nuclei, and cells with secretion vesicles. Metaphase cells were only evident in SP tumor sections. (**A bottom panel**) H & E stained tumor sections generated from ALDH1^+^ and ALDH1^−^ demonstrated the presence of blood vessels, inflammatory/immune cells with heterochromatic nuclei, and cells with secretion vesicles. Metaphase cells were evident in ALDH1^−^ tumor sections. (**B**) non-SP cell derived tumors showed higher numbers of blood vessels (approx. 13) than SP cell derived tumors (approx. 3) and ALDH1^−^ cell derived tumors exhibited more (approx. 7) blood vessels than ALDH1^+^ derived tumors. (approx. 4). (**C**) Histological analyses of tumor sections generated from orthotopically re-transplanted pooled populations of SP, and non-SP derived tumor cells. Sections demonstrated the presence of blood vessels, inflammatory/immune cells with heterochromatic nuclei and secretion vesicles however, sections of re-transplanted pooled SP population derived tumor cells exhibited more blood vessels (≥3) than purified SP population derived tumors (≤3) and re-transplanted pooled non-SP population tumors exhibited fewer blood vessels (5–6) than purified non-SP population derived tumors (9–13). Cells in metaphase were only observed in re-transplanted pooled SP cell derived tumors. Original magnification, 400×. * = blood vessels; I/I = Inflammatory/immune cells with heterochromatic nuclei; SV = secretion vesicles; M = Cell in metaphase.

In addition to tumor growth, the morphology of the tumors was evaluated. Initially, the characteristics of 24 tumors *i.e.,* an average of 6 from each group were evaluated in a blinded fashion on hematoxylin and eosin stained mid-plane of stained sections of each tumor by three investigators. The mid-tumor sections were characterized as to area and extent of central necrosis. All tumors were undifferentiated adenocarcinomas (Figure 6A). The tumors fell into two broad categories: large size (>3 mm in diameter) with metaphases and central necrosis, moderate to high blood vessel densities (Figure 6B) *i.e.,* about seven or greater blood vessels per high power field (400×) with moderate diffuse inflammatory/immune cell infiltrates and with limited protein secretion vesicles, versus smaller (<3 mm in diameter) tumors with minimal necrosis, lower blood vessel densities *i.e.,* about four or fewer blood vessels per high power field (400×), with moderate to high content of secretory vesicles, without significance evidence of inflammatory/immune cell infiltrates. Note that microvessels have been reported to predict metastatic status in breast cancer [41]. Since this is a syngeneic model, the stimulus for accumulation of inflammatory/immune cells was unclear, unless release of molecular components of apoptotic cells was a factor. Multiple elongated/spindle cells were evident in different extents and at various sites of many tumor sections. Morphologically these resembled myoepithelial cells but could be cells in hypoxic areas of the tumors that were attempting to access oxygen from adjacent blood vessels or exhibiting epithelial to mesenchymal transition. The co-relation of morphological characterization of tumors e.g., blood vessel density with factor production requires further investigation.

At this point, the blinding code was broken. The former group of larger tumors was generated by SP and non-SP cells (Figure 6A top panel). The primary difference between these two types of tumor was blood vessels densities, which surprisingly (based on growth rates), were higher in non-SP versus SP tumors. Cells in metaphase were more evident in SP (3.0±1.5) than non-SP (0.7±0.5) tumors, and these data correlated with the growth of tumors. The second group of smaller tumors was generated by ALDH1^+^ and ALDH1^−^ cells (Figure 6A bottom panel). The only differences between ALDH1^+^ and ALDH1^−^ was observed in blood vessel densities and numbers of secretion vesicles with proteinaceous product. ALDH1^+^ tumors exhibited fewer blood vessels and secretion vesicles than ALDH1^−^ tumors. These data demonstrated that not only does the number of cells required to form tumors differ between breast cancer cells with or without stem phenotypes, but also the size, relative to time of growth and morphology of the tumors differed between SP, non SP versus ALDH1^+^, ALDH1^−^ tumors. Cells in metaphase were more evident in ALDH1^+^ (1.3±0.5) than ALDH1^−^ (0.7±0.5) cells. These data indicate that *in vitro* heterogeneities evident in Cl66 murine breast cancer cells, with or without stem cell phenotypes, extended to the morphologies of tumors that these cells generated *in vivo*.

### Demonstration of *in vivo* Self-renewal by Re-transplantation of Cells from Primary SP, Non SP, ALDH1^+^ and ALDH1^−^ Tumors

An *in vivo* re-transplantation assay within an initial set of experiments was performed. Pooled populations of 1000 cells and with 200 cells in a second set of experiments of primary tumor cells derived from SP and non-SP cells showed similar growth characteristics and similar sized tumors. However, a re-transplantation assay with pooled cells of primary tumors formed by ALDH1^+^ and ALDH1^−^ cells did not produce significant tumor growth (data not shown). Analysis of tumors formed by purified SP cells *i.e.* initially 100% SP cells, showed a rapid loss of the majority of cells with the SP phenotype and at necropsy after 3 weeks, these tumors only had the SP cell content of unsorted cells (0.06%). In contrast, tumors formed initially from 100% non-SP cells, i.e. 0% SP cells, acquired a significant content of SP cells (0.29%) by the time of necropsy at the 3rd week. This indicated that the SP phenotype was pliable *in vivo* and was influenced by currently undefined factors. Other studies have shown that the stem cell versus non-stem cell content of tumors can be influenced by IL6 secretion [42] and increased by hypoxia [43]. These are candidate mechanisms, that could be involved both at the time of the initial injection of the cells into the mammary fat pad (and could depend on the number of cells injected) and/or subsequently, on areas of necrosis within the growing tumors which might increase as tumor size increases or be influenced by blood vessel densities at the site of the tumor. These important issues require additional analyses.

When we analyzed the H & E stained tumor sections (Figure 6C), of pooled SP cell generated tumors, we noted they exhibited more blood vessels than the original SP cells. However, pooled non-SP generated tumors showed fewer blood vessels than the original non-SP cell tumors. In addition, the pooled SP tumors had a lower content of SP cells compared to the pooled non-SP tumors. This implicates the tumor cell types in the stimulation of blood vessel formation which, in turn, prompted an evaluation of the soluble factors produced by these tumor cells. In addition, or alternatively, these data suggested that there might be a differential response to hypoxia by SP versus non-SP cells, with SP cells being more resistant to the effects of hypoxia.

### Increased Levels and Numbers of Cytokines/Chemokines were Produced during Interaction of Clone 66 with MS-5

**Figure 7:**
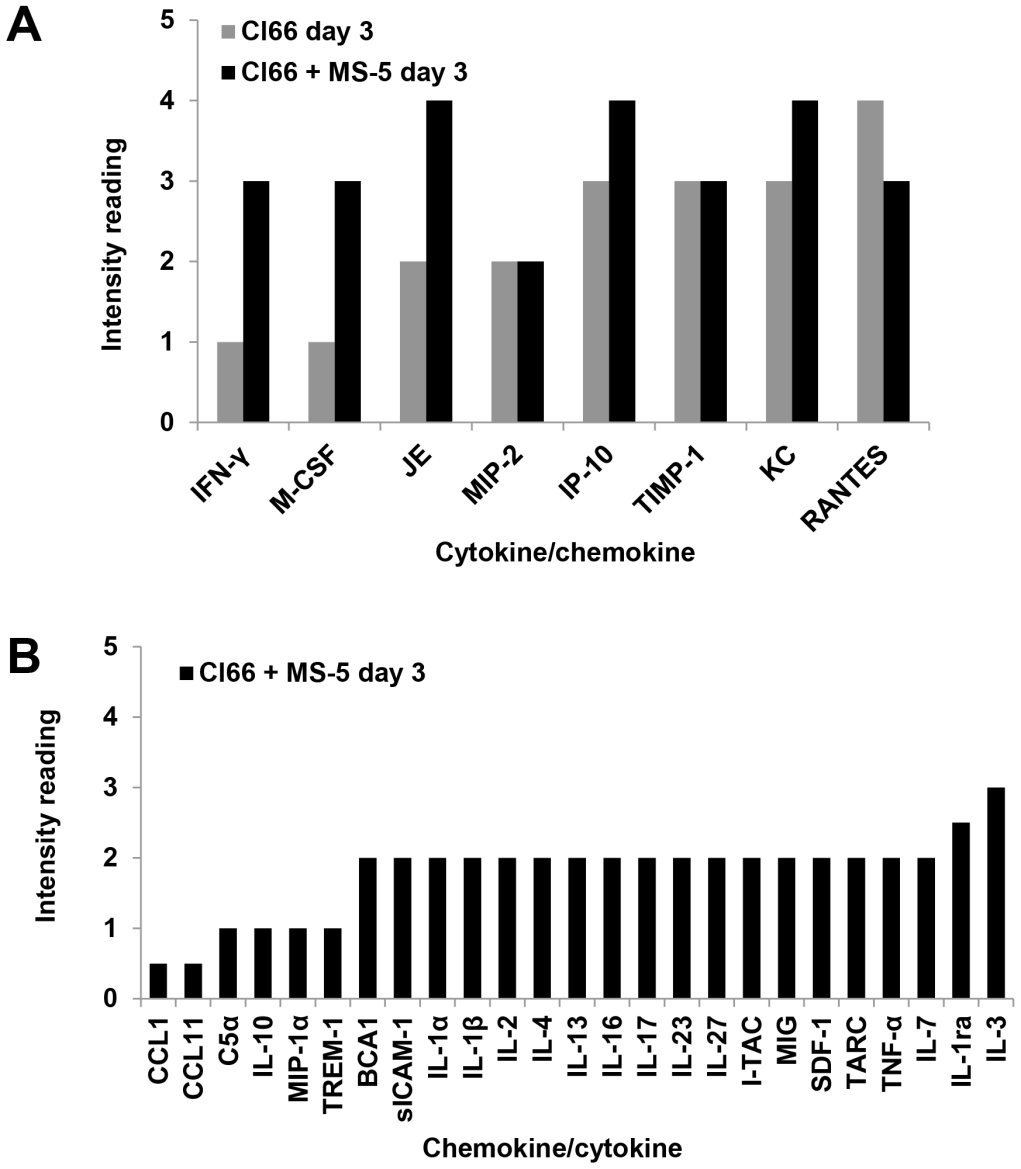
Levels and numbers of cytokines/chemokines produced by Cl66 alone and co-cultured Cl66 with MS-5 stromal cells. Media were collected at day 3 from Cl66 cells alone and co-cultured Cl66 with MS-5 stromal cells; and subjected to analysis of the cytokine/chemokine profiles. (**A**) Histograms show the production of chemokine(s)/cytokine(s) by Cl66 cells alone and changes influenced by co-culture with MS-5 stromal cells. (**B**) Histograms demonstrate the production of chemokine(s)/cytokine(s) during *in vitro* interactions of Cl66 with MS-5 stromal cells.

A role for cytokines/chemokines in growth and metastasis of breast cancer [42], [44] and other cancers [45]–[47] has been described. We observed characteristic differences in tumors generated from SP, non-SP, ALDH1^+^ and ALDH1^−^ cells transplanted to the mammary fat pad of syngeneic Balb/c mice and the pliability of these phenotypes *in vivo*. Differences in blood vessel densities were observed. Moreover, SP and non-SP tumors showed a variable presence of inflammatory/immune cells that suggested factor production by these two populations might differentially attract inflammatory/immune cell populations. In addition, we also observed that fewer SP cells were needed to form tumors than ALDH1^+^ cells, also suggesting either the presence of more cells with stem phenotypes in SP populations or heterogeneities related to the levels of factors/cytokines/chemokines produced by the differing cell populations, which could promote tumor cell growth in the microenvironment. As the *in vitro* sphere forming capacity of SP and ALDH1^+^ cells was found to be the same (4–5%), we investigated the production of cytokines/chemokines by these cells without and with the presence of mesenchymal stromal cells. The intrinsic production of cytokines by Cl66 was limited to eight molecules (Figure 7A). Of these, RANTES was highest, with IP-10, TIMP, and KC intermediate high, followed by mid-level JE, MIP-2; and low levels of IFN-γ, M-CSF (Figure 7A). Co-culture of Cl66 with stromal MS-5 increased the levels of multiple chemokines/cytokines (Figure 7A) and the production of other chemokines/cytokines (Figure 7B) indicating that one consequence of the interaction of Cl66 murine breast cancer cells with stromal MS-5 cells was an increase in cytokine production. Note, in these co-culture experiments, that the source (tumor cells or stromal cells) of these chemokines/cytokines was not defined in this study. Overall, RANTES was high in both Cl66 cells alone and in association with stromal cells. IP-10, KC and JE levels were also highly elevated in association with stromal cells.

Attempts to evaluate cytokine production by sorted stem cell populations were challenging because these populations are rare, and it proved to be difficult to obtain sufficient levels of protein for reliable assay. Although cell numbers could be amplified by growth in culture, these cells progressively lost their stem cell phenotype with time and thus reverted to the composition of unsorted cells.

## Discussion

Various investigators have isolated CSCs, which are proposed to be therapy resistant [48], [49] and responsible for relapse of the tumor following remission after therapy [50] as well as metastases [51]–[53], employing different phenotypes including side population (SP), CD44^high^CD24^low/neg^, ALDH1^+^, CD34^high^, CD133^high^, and CD49f^high^ in either murine or human breast cancers [5], [22], [23], [54]–[58]. The majority of these different populations have not previously been directly compared for functions. However, when comparisons of the functions of such cells were undertaken in this study, using Cl66 murine breast cancer cells, we observed that breast cancer cells with various stem cell phenotypes were present in various proportions in the same cell population. This comparison itself indicated that the stem cell compartment was likely heterogeneous. However, overlap was difficult to address directly in an analysis of multiple stem cell phenotypes, because of challenges associated with spectral overlaps of the fluorescent markers *e.g.,* SP and ALDH1^+^. However, based on differing numbers of such cells in any population of cells, it is very possible that they represent functionally different, although potentially overlapping, cell populations. This question was addressed in these studies.

Prior studies have demonstrated that the ability of breast cancer cells to form mammospheres *in vitro*, especially, secondary and tertiary mammospheres, depends on the presence of self-renewing, gland-reconstituting cells with a stem phenotype within the population [31], [36]. Further, the use of mammosphere assays to assess the presence of stem cells in a population of mammary epithelial cells (MECs) has been further validated by the fact that a single murine mammosphere can regenerate an entire mammary ductal tree when transplanted into a cleared mouse mammary stromal fat pad [31]. We observed that cells with stem phenotypes formed more spheres than non-stem type cells. Our results showed that SP cells formed more and bigger spheres than other cells with stem phenotypes; and this suggested that the SP cell population contained a greater number of cells with stem cell characteristics. When we analyzed the formation of secondary spheres, we observed no sphere formation by non-SP, ALDH1^+^ and ALDH1^−^, CD34^low^ populations. In contrast, we observed sphere formation with SP, CD34^high^ CD49f^high^, and CD49f^low/neg^ suggesting self-renewing cells in SP, CD49f, and CD34^high^ populations. The formation of secondary spheres in both CD49f^high^, and CD49f^low/neg^ populations indicated that this phenotype was not a good stem versus non-stem cell distinguishing marker.

Based, in part, on normal tissue systems, colony forming efficiency is likely a marker of more differentiated, progenitor cells than stem cells [59]. We observed that the colony forming efficiency was higher (>40%) from ALDH1^+^ cells than SP cells (<40%), which suggested that ALDH1^+^ might represent an enriched progenitor cell population. This would correspond also to their lower ability to generate secondary and tertiary spheres. Further, anchorage–independent growth is a trait commonly used to determine the oncogenicity of cells *in vitro* [60]. Obvious increases in soft agar large colony formation were observed with cells with stem phenotypes compared to non-stem type cells with the exception of CD49f^pos^ cells. Greater numbers of soft agar colony formation by ALDH1^+^ cells than SP cells might also suggest the quiescence nature of SP cells. The differences in SP and non-SP phenotypes might be associated with gene transcription expression or epigenetic differences that may play important role(s) in determining stem cell phenotypes [61].

The coordinated interactions of epithelial cells with their stroma is fundamental to controlling their growth and differentiation in normal and pathological situations [62]. We therefore, sought to identify cell populations with or without stem phenotypes that had the highest tumorigenic potential in the stromal environment of the mammary fat pad by *in vivo* orthotopic tumor formation in syngeneic recipients. When we investigated *in vivo* tumor formation by orthotopically transplanting cell populations with or without stem phenotypes into mouse mammary fat pads, we observed that fewer cells with a stem cell phenotype were needed for tumor formation than “non-stem” cells. Further, the SP population was the most tumorigenic as it produced tumors when as few as 50 cells but not when only 25 cells were transplanted. However, when large enough numbers (*e.g.* 6000 cells) of cells with stem cell phenotypes (SP) as well as non-stem cell phenotypes (non-SP) were transplanted, they also formed tumors, suggesting quantitative rather than qualitative differences in tumorigenicity. Also fewer SP cells were needed to form tumors than ALDH1^+^ cells confirming the conclusions of the *in vitro* studies that the SP population has a greater content of cells of a self-renewing tumorigenic stem cell phenotype than ALDH1^+^ cells that appear to have properties expected of more differentiated stem or progenitor cells. Comparisons of *in vivo* tumor growth showed that tumors formed by SP cells grew faster than non-SP, ALDH1^+^ and ALDH1^−^ cells. Re-transplantation of pooled populations of SP and non-SP cells suggested that the non-SP cell population in mammary fat pad induced more blood vessels than SP cell tumors. When we analyzed non-SP tumors using FACS analysis, we observed that the number of SP cells increased sharply with time from 0% to 0.29% within non-SP tumors. In contrast, SP tumors showed a decrease of the SP population from 100% to 0.06% suggesting that two populations appear to maintain equilibrium *i.e., in vitro* 100% SP cells eventually generated the same proportion of SP versus non-SP cells present in unsorted cells. Speculatively, tumor hypoxia may regulate this equilibrium, but this remains to be evaluated.

Earlier studies suggested that cells with CD44^high^CD24^low/neg^ phenotype were more metastatic than non- CD44^high^CD24^low/neg^ cancer cells [51]–[53]. In this study, we have not evaluated metastasis of various cell populations with stem phenotypes due to the challenges associated with the detection of metastases when mice are transplanted minimum numbers of cells (*e.g.,* 50) and the size of the primary tumors is restricted by animal welfare concerns. This warrants future study.

Histopathologically, it was evident that ALDH1^+^ and ALDH1^−^ cells had similar inflammatory/immune cell infiltration and necrosis however; a difference was that ALDH1^+^ tumors exhibited fewer blood vessels, and secretion vesicles with proteinaceous product than ALDH1^−^ tumors. SP and non-SP tumors were similar as regards to the inflammatory/immune cell and central necrosis. However; blood vessels and the presence of vesicles with secretory products were more evident in non-SP than SP tumors. Further, fewer blood vessels were evident in SP tumors than ALDH1^+^ and ALDH1^−^ tumors. The re-transplantation assays showed that blood vessel density in re-transplanted pooled non-SP generated tumors was lower than originally transplanted non-SP cell tumors but more than originally transplanted SP cell tumors suggesting that cells with a non-SP phenotype are capable of inducing more blood vessels for tumor growth and survival whereas SP cells can grow and form larger tumors with minimal blood vessel density, *i.e*., SP tumor cells are relatively more resistant to lack of blood supply and hypoxia. We speculate this reflects relative angiogenic factor production (non-SP cells) versus relative self-renewal activities (higher for SP cells), but this requires more detailed evaluation.

Evidence indicates that soluble factor(s) such as IL6 produced by non-stem type cells have the capacity to convert non-stem type cells to stem cell-type cells [42]. Also, IL6 and IL8 appear to be important in breast tumor growth [40]. Because of this and because of the differences in tumor BVD, we analyzed the various cell populations for the production of soluble factors. The tumorigenic behavior of various cell populations with stem cell versus non-stem cell phenotypes could be a characteristic related to heterogeneities in levels of cytokines/chemokines produced by the differing cell populations and which influence growth of tumor cells in their microenvironment(s). The data from this study indicated that there were heterogeneities in functions of cell populations with various stem phenotypes isolated from the same population of breast cancer cells of Cl66 which may be context dependent and based on stromal environments, factor production or receptor expression, and signaling pathway utilization. Co-culture of Cl66 with stromal MS-5 cells increased the levels of multiple cytokines. However, without stromal MS-5 cells, the intrinsic production of cytokines was limited to eight. Of these, RANTES was the highest, with IP-10, TIMP, KC intermediate followed by mid-level JE, MIP-2 and low levels of IFN-γ, M-CSF. A recent study of MSC (mesenchymal stromal cells) exposed to hypoxia showed that hypoxia increased the levels of several cytokines in the secretome [63], [64].

Evidence suggests that RANTES (CCL5), one of the murine homologues of IL8, has angiogenesis-related activities [65]–[68] and CCL2 (JE or MCP-1) is indeed a potent angiogenic chemokine [66], [69]–[73] that acts by increasing the presence of tumor associated macrophages (TAMs) at breast tumor sites, possibly by elevated release of angiogenic factors and by acting directly on endothelial cells to promote angiogenesis. Many other cytokines that are associated with angiogenesis such as, IP-10, IFNγ, MIP-1α, IL1α, IL-2, IL-4, IL-10, IL-13, IL-17, I-TAC, MIG [74] are found to be up-regulated after Cl66 and stromal MS-5 interactions indicating the possible important contribution of angiogenesis *in vivo*. RANTES was also proposed earlier to be considered as a biomarker for disease progression in stage IIA breast cancer patients [75] and currently, is being suggested as an inflammatory mediator with pro-malignancy activities in breast cancer [76]. Recent pre-clinical and clinical studies have revealed that RANTES is strongly associated with invasiveness [77] the progression of breast cancer, particularly triple negative breast cancer [78]. The interaction of CCL5 with its receptor CCR5 promotes cancer cell migration under hypoxia [79]. JE specifically attracts monocytes *in vitro* and *in vivo*, but has no detectable effects on neutrophils or lymphocytes. IP-10 (CXCL10) induces lymphocytic infiltration [80]; and may act in a paracrine manner by affecting tumor microenvironment and in an autocrine manner by acting on tumor cells and may play a role in tumor invasiveness and progression [80]. Stromal MS-5 interaction induced up-regulation of MIP-2, MIP1α, RANTES, and TIMP-1 that are associated with tumor progression [74], [81]. SDF-1 is associated with tumor progression and metastasis. A paracrine network of KC (CXCL1) links cancer chemoresistance and its metastasis [82]. We observed similar expression in the level of MIP-2 (CXCL2) and TIMP-1 by Cl66 alone and also in the presence of stromal MS-5 cell. M-CSF (an anti-inflammatory mediator) was found to induce diverse anti-inflammatory types of macrophages, known under the generic term M2 macrophages [83]. A more detailed analysis of the role of soluble factor production in breast cancer growth and metastasis is warranted because these are potential therapeutic targets.

Although co-culture of the tumor cells with stromal cells generally increased cytokine production, the cellular source of these cytokines could not be determined. Also, it was difficult to quantitate cytokine production by specific sorted stem cell populations because of their rarity. Evaluation of these mechanisms by molecular manipulations is a future challenge. Overall, Cl66 murine breast cancer cells isolated based on different stem cell phenotypes as well as non-stem cells exhibit functionally heterogeneous behaviors. It will be important, in the future, to attempt to extend these studies to human breast cancer. However, concerns that the efficiency of tumorigenesis will be influenced by the mismatch between the human tumor cells and mouse microenvironment, as noted in lymphoma [16], currently cannot be circumvented.

## Supporting Information

Figure S1 **Generation of tumor spheres from sorted cell populations of Cl66 murine breast cancer cells.** Tumor spheres were obtained by culturing sorted cells from Cl66 murine breast cancer cells in tumor sphere medium containing EGF and bFGF. Cells were plated at a density of 1000 cells/ml. Micrographs show the tumor spheres formed at 7–10 days. Original magnification, ×40. With Matrigel™ denoted as +M. Addition of Matrigel™ increased the size of spheres with SP, ALDH1^+^, and CD34^high^ when compared with their respective non-stem cell populations.
(TIF)

Figure S2 **Generation of agar colony from sorted cell populations of Cl66 murine breast cancer cells.** Agar colonies were obtained by culturing sorted cells from Cl66 murine breast cancer cells in soft-agar containing complete DMEM media with and without Matrigel™. Phase-contrast images show the agar colonies formed by cell populations with stem and non-stem cell phenotypes after 2 weeks. Original magnification, 100×. With Matrigel™ denoted as +M.
(TIF)
