# Supplementary figures and images for: Heterogeneity of Functional Properties of Clone 66 Murine Breast Cancer Cells Expressing Various Stem Cell Phenotypes
Source: PLoS One. 2013 Nov 12;8(11):e78725. doi: 10.1371/journal.pone.0078725 (PMC3827106; doi:10.1371/journal.pone.0078725)

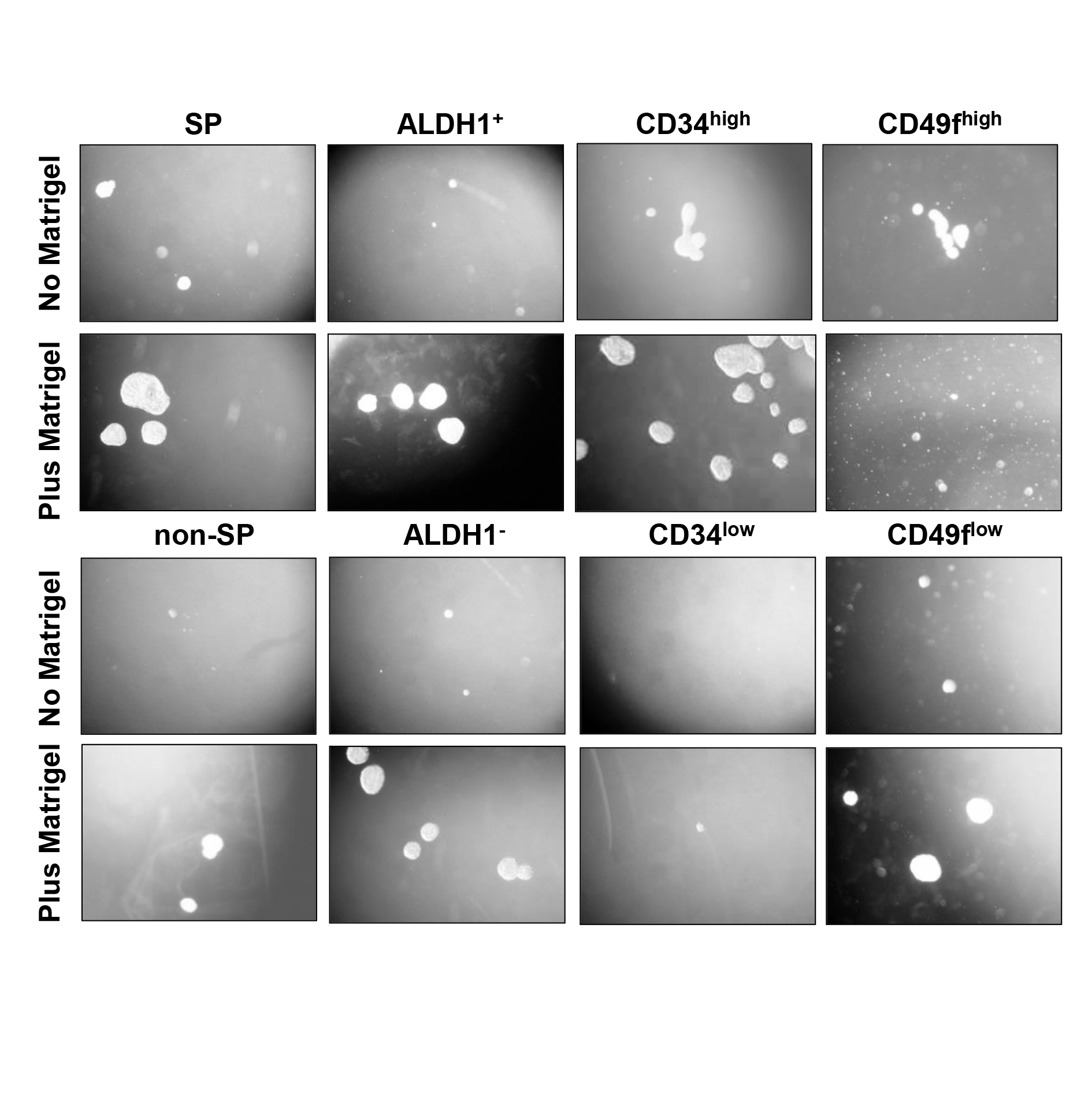

Supplement: Figure S1 — Generation of tumor spheres from sorted cell populations of Cl66 murine breast cancer cells. Tumor spheres were obtained by culturing sorted cells from Cl66 murine breast cancer cells in tumor sphere medium containing EGF and bFGF. Cells were plated at a density of 1000 cells/ml. Micrographs show the tumor spheres formed at 7–10 days. Original magnification, ×40. With Matrigel™ denoted as +M. Addition of Matrigel™ increased the size of spheres with SP, ALDH1+, and CD34high when compared with their respective non-stem cell populations. (TIF) [file pone.0078725.s001.tif]

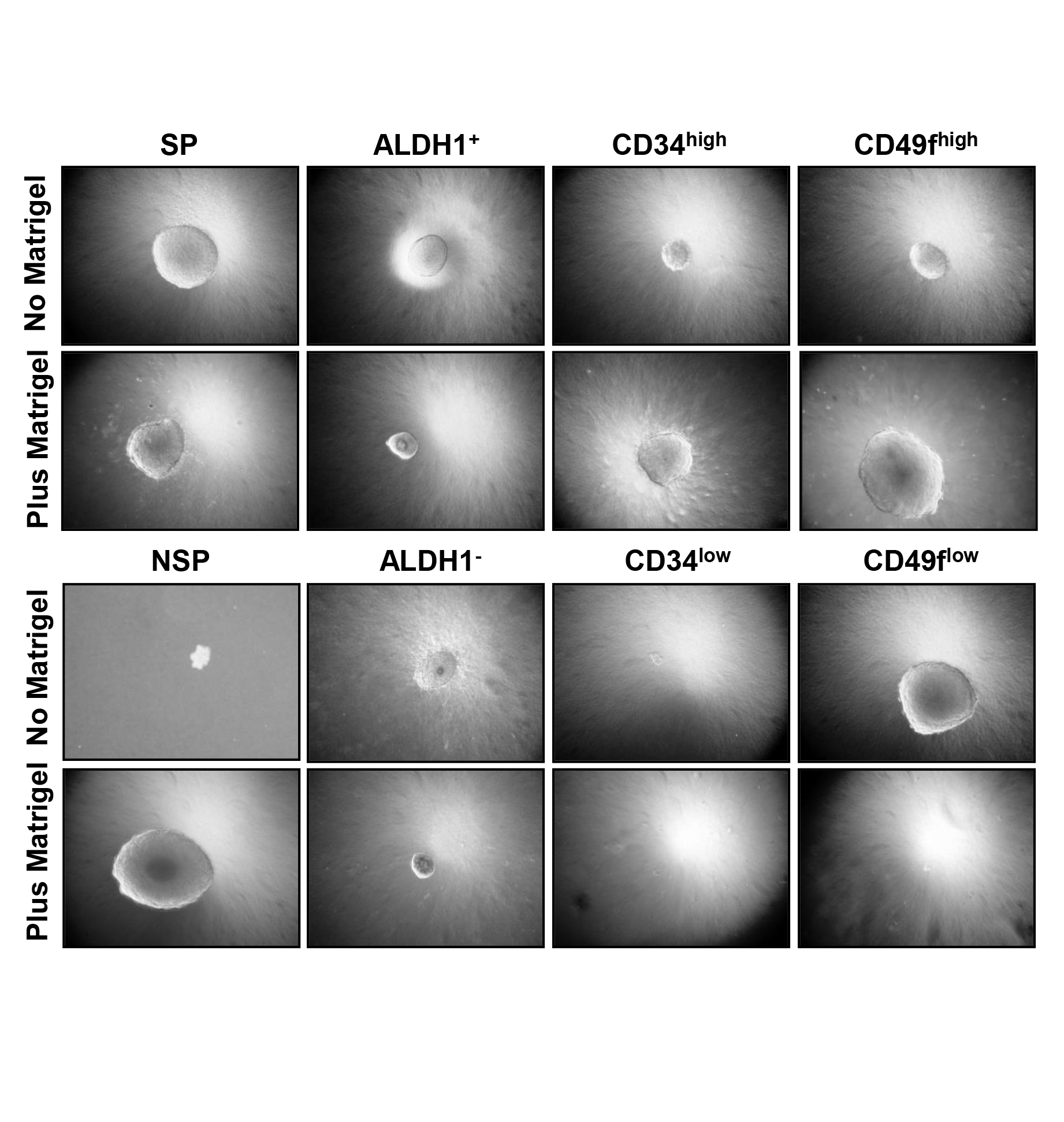

Supplement: Figure S2 — Generation of agar colony from sorted cell populations of Cl66 murine breast cancer cells. Agar colonies were obtained by culturing sorted cells from Cl66 murine breast cancer cells in soft-agar containing complete DMEM media with and without Matrigel™. Phase-contrast images show the agar colonies formed by cell populations with stem and non-stem cell phenotypes after 2 weeks. Original magnification, 100×. With Matrigel™ denoted as +M. (TIF) [file pone.0078725.s002.tif]
